# Doctor of Osteopathy

**Published:** 1902-10

**Authors:** 


### "Doctor of Osteopathy."

WHATEVER we may think of the osteopaths from a professional viewpoint, there is at least one matter with which they are in accord with the medical profession, Mark Twain and the greater part of the church world, and that is their common sense view of the fad of Mother Eddyism in general and christian science in particular. There is a little pamphlet published in the interests of the osteopaths entitled, Osteopathic Success, which in a recent issue brands the seeress of the far east as a fraud and her belief as a fake.

An interesting feature—interesting to the medical mind—of the same issue is that portion devoted to clinic cases from the field, wherein are reported some of the cures of osteopathy by several active practitioners who have been decorated with the title and degree of D. O. Four cases will suffice. The first was a goitre on a man of 40.

His mother also had been suffering with a very large one. Assuring him that there was help for him in osteopathic treatment, I took the case, treating him twice a week, and at the expiration of two months the goitre was cured. The lesion consisted of an anterior condition of the fifth cervical vertebra, and a falling of the clavicle.

Here endeth the first lesson in etiology and pathology.

The second case was that of a man who was suddenly "taken with a very weak and irregular beating of the heart. Osteopathic diagnosis revealed an enlarged condition of the liver and lesions in the pneumogastric nerve." Eight treatments cured the gentleman. Then along came the third wonder: a case of diabetes mellitus in which the characteristic symptoms are reported and where osteopathy shines like a shooting star, for a new appetite is discovered. This patient had an "erotic appetite." Osteopathy is entitled to and deserves all the credit for the discovery of this new symptom in diabetes. Anyway, in spite of his erotic appetite, this patient received nine treatments and went back to work. Twelve other treatments cured him. In this case the lesions "were found in the nerve centers of the liver and kidneys."

The fourth case does not give the location of the lesion; but the "patient was most efficiently helped over that critical period in which collapse seemed imminent and started on a new epoch of life with fifteen osteopathic treatments."

From these cases osteopathy appears to be a case of "D.O., D.O., my huckleberry, D.O."

---

The German courts have rendered a decision of great importance to the medical profession in the case of a Nuremberg physician who was arrested on a criminal charge for neglecting to notify the authorities of the death and mutilation of a newborn infant. The physician was called to attend a woman who was seriously ill. In his examination he discovered that she had recently given birth to a child and he found the mutilated remains of the infant in a vessel under the woman's bed. Apparently the child had been dead but a short time. He deemed it his duty to remain silent regarding the criminal aspects of the case and went on with the treatment of the woman just as though he had not discovered the child's body. In some way the facts became noised about and when the police made an investigation they learned the physician's connection with the case and his knowledge of the death of the infant and he was placed under arrest charged with what in this country would be equivalent to being an accessory. On trial he set up as his defence that he had acquired the knowledge of the alleged crime in his professional capacity; that had he not been in confidential relation with the mother as her physician, he would not have known of the existence of the child's body; that none but a physician could have known of the woman's condition and then only could he receive the facts as a matter of professional confidence. The courts held that his position was correct and within the law; that to have made known his discovery would have been to violate the confidential relations existing between physician and patient, and he was discharged from custody after an honorable acquittal.

---

Dr. Sydney A. Dunham, of Buffalo, has secured the beautiful Dunham home of 1392 Amherst Street, which he will occupy as his private residence and keep open for the care and treatment of selected nervous patients. Dr. Dunham has been convinced for sometime of the great need in this vicinity for a strictly private home for certain forms of nervous disease. The environment and surroundings of the house chosen are of the country sort. It is surrounded by an acre and a half of land opposite the Park Meadow and is covered by native maple and oak trees.

Drug and alcoholic habitues are excluded. Patients may have the attendance of family physician while at the home.

All communications should be addressed to Dr. Sydney A. Dunham, office, 239 Delaware Avenue, Buffalo, N.Y.

